# **Identification and mitigation of blood’s interference with the antimicrobial activity of AgNbO**_**3**_ **particles**

**DOI:** 10.1371/journal.pone.0313055

**Published:** 2025-06-24

**Authors:** Cyrus Talebpour, Fereshteh Fani, Marc Ouellette, Marilynn Fairfax, Houshang Alamdari, Hossein Salimnia

**Affiliations:** 1 Department of Mining, Metallurgical and Materials Engineering, Université Laval Faculté des Sciences et de Génie, Québec, Quebec, Canada; 2 Department of Microbiology and Immunology, Université Laval Faculté de Médecine, Québec, Quebec, Canada; 3 Department of Pathology, Wayne State University School of Medicine, Detroit, Michigan, United States of America; Al-Azhar University, EGYPT

## Abstract

The detrimental impact of blood on the antimicrobial activity of AgNbO_3_ particles was identified and investigated. It was observed that the impact is more severe in the case of lysed blood. The same phenomenon also operates in the case of commonly used silver salt, AgNO_3_. The inhibition was shown to be due to hemoglobin, but may be unrelated to the heme moiety. In an attempt to find additives to mitigate the inhibitory effect of hemoglobin, iron ions and the chelating agent, K_2_EDTA, were initially considered as potential candidates. Including ferric iron on the particles was shown to have a marginal effect, but supplying the medium with K_2_EDTA chelating agent, provided a better outcome for countering the deleterious impact of hemoglobin on AgNbO_3_ activity. These findings may be relevant for adapting the silver compounds to applications such as wound dressings, where silver’s antimicrobial action would have to take place in a blood containing environment.

## 1. Introduction

Silver compounds, including silver salt AgNO_3_, silver nanoparticles, and silver zeolites have been intensively studied as broad-spectrum and relatively safe replacements for antibiotics in diverse biomedical applications [1–4]. Although different mechanisms of action have been proposed to explain the antibacterial activity, there is general agreement on the central role of silver ions, which become available through dissociation, corrosion, or ion exchange [5]. The elution of ions from relatively stable silver nanoparticles, which occurs via the intermediate formation of Ag_2_O on the surface of the nanoparticles by dissolved oxygen or hydrogen peroxide formed as metabolic product of a nearby microorganism, is thought to have a central role in antimicrobial action [6]. Release of silver ions results in their depletion the loss of antimicrobial activity over time and may compromise biocompatibility due to potential risks when the compound is used in clinical applications, such as wound-dressing or medical implants. To achieve a more biocompatible alternative we fixed the silver atoms in a stable crystal structure (AgNbO_3_) to protect it from corrosion and dissolution [7]. Since untreated AgNbO_3_ does not have noticeable antibacterial activity, we came up with a series of thermo-mechano-chemical processes to successfully activate AgNbO_3_. This top-down procedure, previously developed and optimized by our research group, transforms the AgNbO_3_ into nanostructures with altered physicochemical properties enabling antimicrobial activity where Ag^+^ release does not play a central role. This behavior was reported in one of our previous publications [8]. There, we demonstrated that the level of Ag^+^ release from AgNbO_3_ at its minimum inhibitory concentration (MIC) was substantially lower than that of the reference AgNO_3_ salt at MIC, indicating that the quantity of Ag^+^ release from AgNbO_3_ is not sufficient by itself to lead to bacterial death. Moreover, we have demonstrated that AgNbO_3_, when incorporated into Polymethyl methacrylate (PMMA) bone cement, exhibited strong antibacterial activity towards *Staphylococcus aureus* and *Pseudomonas aeruginosa* without causing cytotoxic effects on human THP-1 cells, supporting their biocompatibility in clinical applications [9]. The resulting nanostructured particles may be a suitable replacement for antibiotics or conventional silver in areas such as antimicrobial dental implants [10], bone cement [11], wound dressings [12], and hip and knee implant devices [9,13], thanks to their durability, corrosion resistance, and negligible levels of toxicity to the surrounding tissue.

Most of the reported activity assays of silver compounds reported by others have been performed in culture media without human blood or its components. Thus, their results are not necessarily transferrable to complex biological environments where the presence of interfering material may affect the antimicrobial activity of Ag^+^ in vivo [14,15]. This point has been discussed by Croes et al with respect to the poor bactericidal effects of Ag-based coatings in bone infection models [16].

Upon the recognition of the reduced activity of silver ions under physiological conditions, strategies for addressing it were also investigated. For instance, Vasiliev et al have shown that addition of CuSO_4_, resulting in the release of copper ions, can counter the impact of serum proteins on the activity of AgNO_3_ [17]. This is an instance of synergism of the silver/transition metals, which has been demonstrated to result in increases of up to 8-fold in antimicrobial activity against certain bacterial species [18,19].

The above observations highlight the importance of identifying and countering the effects of interfering biological materials in order to establish the suitability of a target antimicrobial for a prospective application. In this study, we investigate the potential inhibitory impact of blood on the antimicrobial activity of the AgNbO_3_ particles. We first investigated this effect by monitoring the inhibition of the antimicrobial effect of the particles in the presence of whole and lysed blood. Based on the results obtained here, we next determined the adverse effects of suspected blood components. Finally, we shifted our focus to mitigating the impact of implicated blood components by selected additives, including iron ions and the chelating agent, K_2_EDTA.

## 2. Methodology

### 2.1 Preparation and characterization of nanostructured antimicrobial particles

The antimicrobial particles have a formula of AgNbO_3_ and were synthesized by employing the activated reactive synthesis (ARS) procedure. In this method, the perovskite oxide is synthesized from metal-oxide precursors by high-temperature heat treatment. The raw materials Ag_2_O (Sigma-Aldrich Corp) and Nb_2_O_5_ (Inframat® Advanced Materials LLC), with the weight ratio of 1 g to 1.147 g respectively (for each g of Ag_2_O, 1.147 g of Nb_2_O_5_ powders), were mixed in a hardened steel crucible with high energy ball milling for 10 minutes. The mixture was transferred to a ceramic crucible and placed in a furnace where it was gradually heated at a rate of 5°C/min until the formation temperature, 1000°C, was reached. The mixture was kept at this temperature for about 4 h and was gradually cooled down at a rate of 10°C/min to room temperature. The powder was subjected to high energy ball milling. The milling process was carried out using the 8000D Mixer/Mill® (SPEX SamplePrep, LLC) in which 7 g of material was agitated at 1060 cycles per minute for a duration of 90 min. The apparatus system also contained a supporting crucible and three milling balls. The crucible chosen was the 8001 hardened steel vial set, which contained a vial body and cap liner. One 6.35 mm, and two 12.70 mm steel balls were used for milling. The powder from the high energy ball milling step was subjected to a low energy attrition mill. In this step, approximately 40 g of powder from multiple batches of the previous step (agglomerates) were added to a crucible containing hundreds of steel beads of 4.5 mm in diameter, which were made to rotate at 90 rpm by Szegvari Attritor System Type E Model 01-STD (Union Process, Inc.). To this, 10 mL of water was added and the attrition process was performed for 120 min. At the end of the operation the beads are rinsed with deionized water and the residue thus obtained was dried inside an oven with a temperature of 150°C overnight. As reported in our prior publication, the particles had a specific surface area of 7.9 m^2^/g and the average particle size was 438 nm. The silver release rate of the particles was less than 1% of its total mass after 35 days of storage in water. The physical transformation of the AgNbO_3_ particles throughout the synthesis process is presented in S1 Appendix.

A second batch of nanostructured AgNbO_3_ particles with substitutional doping of Fe_2_O_3_ was synthesized using the same ARS procedure. The only difference was to mix the raw materials Ag_2_O, Nb_2_O_5_ and Fe_2_O_3_ (Sigma-Aldrich Corp) with the weight ratio of 1 g to 1.090 g to 0.0345 g respectively to achieve AgFe_0.05_Nb_0.95_O_3_.

### 2.2 Characterization of nanostructured antimicrobial particles for surface composition

The surface composition of the particles was interrogated by photoelectron spectroscopy (XPS) with an analysis depth of ~10 nm and surface area of 100 µm × 100 µm. The analysis was carried out using the PHI Quantes X-Ray Photoelectron Spectrometer. The survey spectra and the high-resolution spectra were acquired using the monochromatized Kɑ line of standard aluminum (hv = 1486.6 eV) with charge compensation. Photoelectron detection was performed at a take-off angle of 45^o^ with respect to the sample surface for both survey and high-resolution spectra. High resolution C1s spectra were recorded and calibrated at 285 eV for the analysis. The analysis chamber was set to vacuum pressure at 10^−10^ mbar. The analysis of the results was performed using the open source CasaXPS software and the appropriate elemental library for the XPS machine was used to apply the correct relativity sensitivity factor for each atomic level.

### 2.3 Bacterial strain and culture conditions

The bacterial strains used in this study were *Escherichia coli* ATCC # 25922, *Klebsiella pneumoniae* (ATCC 700603) and *Pseudomonas aeruginosa* (ATCC 27853). Cell stocks in 50% glycerol were taken from a −80°C freezer. After thawing, 30–50 µL was transferred to a Tryptic Soy Agar (TSA) (with 5% sheep blood) plate (the P1 plate), inoculated by standard streaking, and incubated at 37°C overnight under aerobic condition until colonies were visible. A well-formed, representative colony from the plate was picked and inoculated into 3 mL of Tryptic Soy Broth (TSB) and incubated at 37°C with 150 rpm shaking for 3 h. Then, 1 mL aliquots were transferred to 2 mL sterile Eppendorf tubes and centrifuged at 8000 rpm for 8 min in a microcentrifuge. The harvested cells were resuspended in 1 mL TSB and 0.5 mL was used for OD_600_ measurement. Using an in-house OD_600_ vs cell count correlation database, a cell suspension of 1.5 × 10^8^ CFU/mL was prepared. The suspension was then diluted 1/100 in TSB to a final concentration of 1.5 × 10^6^ CFU/mL.

### 2.4 Antimicrobial activity on different agar media

Three types of agar plates obtained from Becton Dickinson, including Muller Hinton Agar (MHA) plates, Blood Agar Plate (BAP) and Chocolate Agar Plate (CAP), were used to assess the impact of their composition on the antimicrobial activity of microbial cells. First, on the back of the plate guiding circles were marked to facilitate the two approaches described below.

1) A 5 µL of aliquot of particle suspension, containing preselected concentration of particles (1600, 800, 400, 200, 100, 50 and 0 µg/mL), was dispensed at the center of the designated circles. The particle suspension spread spontaneously to a diameter of ~8 mm and was allowed to air dry for approximately 30 min. According to our previous analysis [20], these circles contained 160, 80, 40, 10, and 5 ng/mm^2^ of particles respectively. Then, 1 µL of bacterial suspension, having a nominal concentration of 10^5^ organisms/mL was dispensed in the center of the particle spot. The plates were incubated for 6 h in 37°C, then left at room temperature overnight, and inspected the next day for bacterial growth. The transfer to room temperature slowed bacterial replication, in order to restrict confluent growth, facilitating the ability to distinguish cases of non-inhibition from partial inhibition.
2) Bacterial cell suspension (1/100 dilution in TSB of a 0.5 McFarland) was spread over the entire surface of the plate using a swab that had been immersed in the cell suspension tube. Then 5 µL of AgNbO_3_ dilutions (50–1600 µg/ml) were loaded onto their designated locations on the plate. For a positive control, 5 µL of water was dispensed in the circle at the center of the plate. The plate was incubated at 35°C overnight and then photographed. This test was also repeated to determine the interaction with different concentrations (100, 50, 20, 10, 1, 0.1 mM) of AgNO_3_ as a reference material.

### 2.5 Measuring the minimum inhibitory concentration by the microdilution method

The antimicrobial activity of AgNbO_3_ particles was measured by the broth microdilution method by growing bacterial cells inside a series of wells on a microwell plate containing growth media. Each well was supplied with the concentration of the antimicrobial agent to be tested, in a series of one to two dilutions. Bacterial cells with the target concentration of 10^5^ CFU/mL were dispensed into each well. After overnight incubation, the wells were visually inspected for signs of growth. The minimum concentration of the particles for which no growth was observed was taken as the minimum inhibitory concentration (MIC) value.

### 2.6 Measuring the antimicrobial activity of AgNbO_3_ particles in the presence of interfering materials

The antimicrobial activity of AgNbO_3_ particles was measured by the broth microdilution method by growing bacterial cells in a series of wells on a microwell plate containing growth media having different levels of hemin (Sigma-Aldrich Corp), hemoglobin (Sigma-Aldrich Corp) FeSO_4_ (Sigma-Aldrich Corp) and FeCl_3_ (Sigma-Aldrich Corp). Each well was supplied with different concentrations of the antimicrobial agent, in a serial one to two dilutions. Bacterial cells with the target concentration of 10^5^ CFU/mL were dispensed into each well. After overnight incubation, wells were inspected visually for bacterial growth. After overnight incubation, 10 μL of each well’s content was transferred to a corresponding well on another microwell plate, containing 150 μL of Luria Bertani (LB) media. This second plate was incubated overnight and the wells were inspected the next day for signs of growth by visual inspection. The minimum concentration of the particles for which no growth was observed was taken as the minimum inhibitory concentration (MIC) value. The tests were also repeated for interaction with different concentrations of AgNO_3_ as a reference material, in the cases of hemin and hemoglobin containing media.

### 2.7 Testing the ability of K_2_EDTA to restore the antimicrobial activity of AgNbO_3_ particles

The test was performed in two formats: solid phase and liquid phase. In the solid phase format, a K_2_EDTA (Becton Dickinson) solution having a concentration of 8 mg/mL was prepared. Different amounts of this solution were spread on 3 Chocolate agar plates, which respectively gave 1600, 800 and 400 µg of K_2_-EDTA per plate surface. After air drying, a bacterial cell suspension (1/100 dilution in TSB of a 0.5 McFarland) was spread over the agar surface using a swab immersed in the cell suspension tube. Then 5 µL of AgNbO_3_ suspensions (50–1600 µg/ml) were loaded onto their designated location on the plate. For a positive control, 5 µL of water was dispensed at the center of the plate. Plates were incubated at 35°C overnight and photographed the next day.

In the liquid phase format, the antimicrobial activity of AgNbO_3_ particles was assessed by microdilution antimicrobial susceptibility testing in six different media, including MHB (Becton Dickinson), with and without 400 µg/mL of K_2_EDTA, MHB with 5% lysed horse blood (Muller Hinton Fastidious Broth) purchased from Liofilchem (with and without 400 µg/mL of K_2_EDTA), and a filter-sterilized MHB containing hemoglobin at a final concentration of 6 mg/mL (with and without 400 µg/mL of K_2_EDTA). For each medium, each well of a row in a microwell plate was supplemented with different concentrations of AgNbO_3_ particles, in serial one to two dilutions. Bacterial cells with the target concentration of 10^5^ CFU/mL were dispensed into each well. The test was performed on three replicate rows. The plate was incubated overnight and was visually inspected the next day for signs of growth. The minimum concentration of the particles for which no growth was observed was taken as the minimum inhibitory concentration (MIC) value.

## 3. Results and discussion

As the main theme of the present work relates to the detrimental impact of blood on the antimicrobial activity of AgNbO_3_ particles, we present a brief description of these particles and their antimicrobial activity in growth media such as Tryptic Soy Broth (TSB) based on our published results [7,8]. AgNbO_3_ particles were suggested to alleviate two issues associated with the widely studies silver nanoparticles (AgNPs): Firstly, despite intensive and extensive research on AgNPs, challenges regarding the cost-effective synthesis and the colloidal stability of these agents are far from over [21]. Secondly, when AgNPs are exposed to physiological media, the formation of silver oxide on their surfaces initiates dissolution and releases silver ions until the silver oxide complex is completely dissolved [22]. This process, though undesired in the context of the antimicrobial activity depletion and clinical toxicity, cannot be eliminated by surface passivation approaches as it is recognized that the antibacterial activity of AgNPs is related to the Ag^+^ release [23,24]. These facts highlight the need for more robust and cost-effective alternative for AgNPs in the form of nano-structure particles. In one approach, silver cations are trapped in an ion-exchanged zeolite [25]. However, as it has been reported that the procedure is reversible, meaning that a zeolite which uptakes cations from a solution can release them if it is put in a solution where the cations concentration is lower than that of the initial solution [5]. As another approach, we tightly incorporated silver atoms in the corrosion resistant perovskite crystal structure of AgNbO_3_ and subjected the compound to a series of mechanical treatments to greatly enhance increased its antimicrobial activity to the level of the reference Ag_2_O particles while keeping its relative silver release to the ambient lower by at about two orders of magnitude.

The niobate component (NbO_3_) of the compound contributes to unusually high dielectric constant and ferroelectric properties of AgNbO_3_ particles. Previously, we speculated that these may promote electrical interactions, leading to better contact between microbial cells and nanoparticles. However, the hypothesis was rejected based on experimental observations indicating lack of antimicrobial activity in CaCu_3_Ti_4_O_12_, with extremely large dielectric constant, or in the ferroelectric LiNbO_3_, with high spontaneous polarizations. Thus, the niobate component has no significant contribution in the antimicrobial activity of AgNbO_3_ particles [7]. It simply provides higher stability and corrosion resistance to the nanoparticles as indicated by reduced silver ion release in extremely basic, acidic, saline and bleach environments (See S2 Appendix). The reduced silver release rate in these environments, as compared to release in water, provides evidence that AgNbO_3_ antimicrobial activity is likely due to surface-mediated interactions rather than bulk dissolution. Indeed, the physical transformation mediated by ball milling (see Fig A of S1 Appendix) is crucial for their antimicrobial activity [7]. This may be explained by nearly 100 × decrease in average crystallite size (from > 500 nm down to 14 nm) and increase in specific surface area (from 0.13 m^2^/g to 7.0 m^2^/g) and widening of light absorption spectrum as indicated by strong darkening of the particles seen in Fig A of S1 Appendix. These changes were correlated with ~100-fold increase in the antimicrobial activity [7], implying that the surface chemistry of the nanoparticles is a major determinant in its interaction with biological material.

We have illustrated broad-spectrum antimicrobial activity of AgNbO_3_ nanoparticles against 16 species of pathogenic bacterial and fungal species (clinical isolates) and have presented the results in S3 Appendix. Though the exact mode of this antimicrobial action remains unclear, at a phenomenological level, the particles cause drastic morphological changes in bacterial cells leading to cellular damage, which are correlated with the production of reactive oxygen species and lipid peroxidation [8].

The main theme of the present work is the potential inhibitory impact of blood on the antimicrobial activity of the AgNbO_3_ particles. We used the surface of agar plates as a “model environment” for our study. This selection provides easy visual inspection for readily detecting inhibitory effects of whole blood or its lysed form by comparing the antimicrobial activity of AgNbO_3_ particles incorporated on different types of plate. Our observations are described in the following subsections.

### 3.1 Antimicrobial activity of AgNbO_3_ particles in the presence of interfering material

The possible impact of blood on the antimicrobial activity of AgNbO_3_ particles is the main focus of the present work as most prospective clinical applications are expected to occur in the presence of blood. The easily replicated approach to quantify this issue is to use three standard plates, one containing no blood (MHA), one containing whole blood (BAP), in which cells are presumed to be intact, and chocolate agar plate (CAP) which contains hemoglobin. The surface minimum inhibitory concentration (sMIC), which in our recent work was shown to be a relevant indicator for applications such as antimicrobial bone-cement [20], was selected as the appropriate quantifier of bacterial toxicity. To measure sMIC, aliquots of serial one to two dilutions of AgNbO_3_ particles are dispensed at delineated spots and are allowed to settle. Then, microbial cells with a nominal count of ~100 are dispensed at each spot. This number is neither too low as to raise the possibility of sampling error, nor too high as to involve overcrowding and make it impossible to discriminate between circles with no inhibition and those intermediate cases when a fraction of cells survived the antimicrobial action and formed colonies.

**Fig 1:**
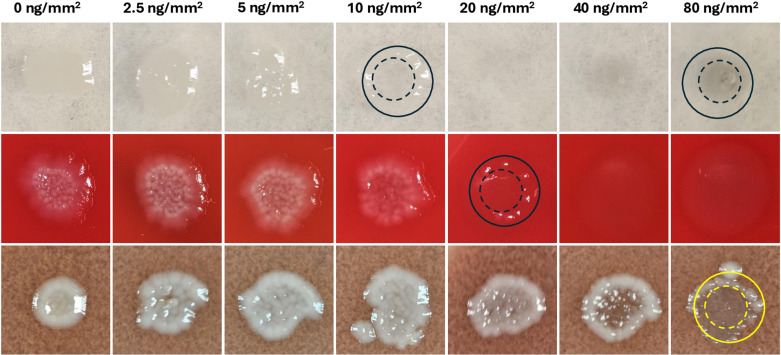
Photographs of the spots of bacterial growth on plates where the particles had been dispensed, and which then received a bacterial load of ~100 CFU. From top to bottom, MHA, BAP, and CAP. The concentric dashed and solid circles drawn on the representative cases, respectively, indicate the regions of plateaued and low AgNbO_3_ particle concentrations (see below).

The results of bacterial growth on three different agar plates, in the presence of varying concentrations of AgNbO_3_ the particles are shown in Fig 1. We observed that in the case of MHA plates, which lacks any blood product, almost no bacterial cells survived at the surface concentration of 10 ng/mm^2^ and considered this concentration as the surface MIC (sMIC), a term introduced by us, and thoroughly defined in our past publication [20]. For BAP plates, having presumably non-lysed red blood cells, the sMIC increased reproducibly from 10 µg/mm^2^ to 20 µg/mm^2^. In the case of CAP plates, having hemoglobin as part of their composition (~0.5% w/v) [26], the sMIC increased to over 80 µg/mm^2^, an 8 × fold increase as compared to the MHA plate.

**Fig 2:**
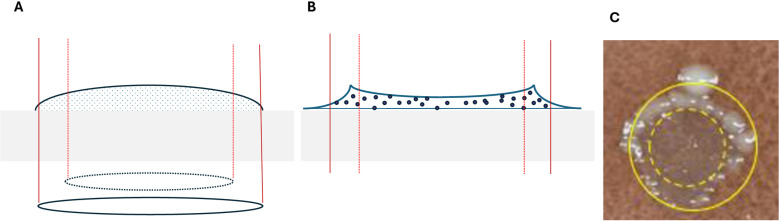
Schematic representation of the distribution at the late drying stage of AgNbO_3_ particles (A) and microbial cells (B) on a hydrophobic gel surface. The two concentric circles, respectively, correspond to the two circles that we had overlaid on the spots of Fig 1, an example of which is presented in (C).

Some comments related to the bacterial colony distribution over the spots, particularly those containing AgNbO_3_ concentrations close to the sMIC value, are warranted. For instance, in the case of the CAP plate, at both 40 µg/mm^2^ and 80 µg/mm^2^ the growth in the central region is partially inhibited, while there is a circumpherential ring of higher growth. This is likely the result of the evaporation of sessile droplets containing non-volatile solutes, resulting in the formation of ring-like residues along the contact line pinning [27]. AgNbO_3_ particles are expected to settle down without significant outward radial displacement due to their high mass density of 6.8 g/cm^3^ [28], thus resulting in a post-drying particle distribution that is a projection of the particle distribution in a flattened drop on the hydrophilic plate. Referring to Fig 2, this distribution has a nearly uniform profile in the central regions, as is actually observed by the darker color of the high intensity spot on MHA (inside the dashed circle at the upper right corner in Fig 1), and reduced surface concentration in the region between the dashed and the solid circles. In contrast, the bacterial cells, due to their small mass density, will relocate preferentially to the peripheral region (between the dashed and solid circles), where the AgNbO_3_ particles are less numerous than in the center region. Therefore, the cells finally settled in the central zone are in contact with more particles and feel their antimicrobial impact to a greater extent.

**Fig 3:**
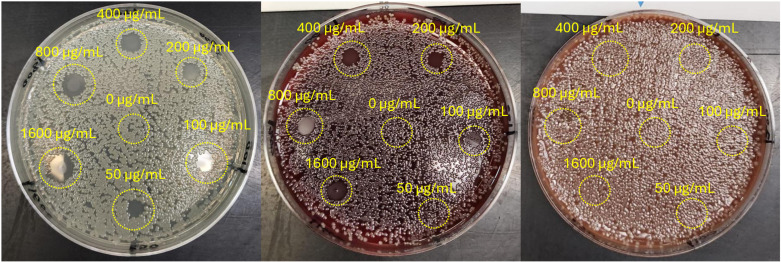
Antimicrobial activity of AgNbO_3_ particles on different plates. From left to right, respectively, MHA, BAP, and CAP. The numbers adjacent to the spots indicate the AgNbO_3_ concentration in µg/mL. Full antimicrobial activity and inhibition of bacterial growth is seen on MHA plate for AgNbO_3_ concentrations ranging from 50 to 1600 microgram/ml. Partial inhibition of AgNbO_3_ antimicrobial activity is seen on BAP plate (MIC of 200 µg/mL; partial inhibition at 100 µg/mL), and complete inhibition of activity on CAP plate for all tested concentrations of AgNbO_3_.

We also studied the dependence of sMIC on the plate composition by a complementary method, where a bacterial cell suspension was spread over the whole area of the three types of plates and left to air-dry. Then, 5 µL of AgNbO_3_ suspension, with concentrations varying by factor of 2-fold in the 50–1600 µg/mL range were dispensed at designated locations on the plates. The post-incubation photos are presented in Fig 3. Clearly, while the MIC of the AgNbO_3_ particles on BAP (200 µg/mL; partial inhibition at 100 µg/mL) was about 2 × higher than on MHA (Full inhibition), which could be attributed to a small amount of red blood cell lysis in that medium, there is a large increase in the MIC on CAP (>1600 µg/mL). The results agree with the sMIC determined via the previous approach, accounting for the more prolonged incubation time. Moreover, to verify whether these findings would translate to other strains that may be found in clinical settings, we have repeated a similar test with *Pseudomonas aeruginosa* and *Klebsiella pneumoniae*, and observed similar results as presented in the supplementary information (See S4 Appendix). This is supporting evidence for the inhibition of AgNbO_3_ particles’ antimicrobial activity on plates containing either lysed blood or hemoglobin.

**Fig 4:**
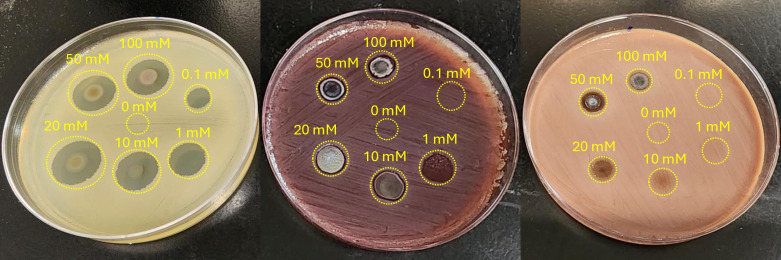
Antimicrobial activity of AgNO_3_ particles on different plates. From left to right, respectively, MHA, BAP, and CAP. The numbers adjacent to the spots indicate the AgNO_3_ concentration in mM. Note that the area of bacterial growth inhibition is diffuse in the case of MHA whereas the diffusion process appears restricted on BAP and CAP. White precipitates found predominantly at the center of inhibition zones of the NaCl rich Mueller-Hinton agar revealed accumulations of AgCl, formed through the reaction of Ag^+^ and Cl^-^ ions provided by the AgNO_3_ and NaCl salts respectively.

A similar shift in the MIC on BAP (1 mM) and CAP (20mM; partial inhibition at 10 mM) plates from MHA (0.1 mM) was also observed when testing for the antimicrobial activity of AgNO_3_ salt, whose mode of action is attributed to silver ions [29]. The test result, which is presented in Fig 4, apart from illustrating a clear increase in the MIC for the cases of BAP and, in particular CAP, also indicates a characteristic feature: We observed an absence of a zone of inhibition beyond the area in which the AgNO_3_ solution was dispensed. Apparently, this observation results from a much slower diffusion of silver ions in a blood containing gel or alternatively, and more likely, to the sequestration of diffusing silver ions, resulting from dissociation of AgNO_3_, by blood [30]. This effect is also seen to a slight degree for the case of AgNbO_3_ at higher concentrations, as seen in Fig 3, which presumably releases small amounts of silver ions, which accounts for only a small part of its mechanism of antimicrobial action [8].

The clear shift in the sMIC of the particles on BAP and CAP with respect to the case of MHA may be explained by difference in biochemicals between MHA and BAP/CAP. In the case of the BAP, we can refer to the study conducted by Mulley et al., which directly described blood-mediated inhibition of silver’s antimicrobial activity, pointing to serum components as the primary inactivating agent [14]. The authors investigated how various biologically relevant compounds in the serum, including albumin, glutathione and chloride ions, interact with silver ions resulting in the formation of silver-protein complexes, silver-thiol conjugates, and silver chloride precipitates, all of which leads to the sequestration of silver ions and the inactivation of their antimicrobial properties.

These identified mechanisms, however, do not fully relate to our investigations on increased inhibition seen in the CAP. To help explain, we should consider that chocolate agar is prepared by adding red blood cells (RBCs) to a molten agar base and then heating the mixture to ~80°C, thereby lysing the RBCs which release their intracellular contents [26,31]. Considering that hemoglobin is estimated to be about 95–97% of the RBC’s dry mass [32], we first point to this molecule as a possible culprit for the inactivation of AgNbO_3_ activity. Muryama et al. first identified that hemoglobin contains thiol groups which are also capable of forming conjugates with numerous heavy metals including silver [33]. More recently, it was found that hemoglobin plays a role not only in the sequestration of silver ions but also of silver nanoparticles. For instance, Zolghardi et al. demonstrated the ability of silver nanoparticles to induce conformational changes in bovine hemoglobin and alter its physiological functions [34]. Mahato et al. found that silver nanoparticles readily form stable complexes with hemoglobin, and that this attachment occurs at a time and concentration dependent rate [35]. Put together, these findings suggest that silver species become trapped within hemoglobin, reducing their availability for antimicrobial action. Finally, it is plausible that the partial inhibition by BAP is likely due to the presence of slight amounts of free hemoglobin resulting from ruptured/lysed RBCs in the medium.

Hemoglobin and hemin were tested for their inhibitory effects on AgNbO_3_ particles because they are more readily available for interaction in the CAPs. We should mention that when the mixture for chocolate agar plates is heated to disrupt the RBC, released hemoglobin may further degrade, releasing hemin, which is an oxidized form of heme [31]. It is therefore of particular interest to use hemin to isolate the effects of the heme group itself, independent of the protein structure of hemoglobin.

**Fig 5:**
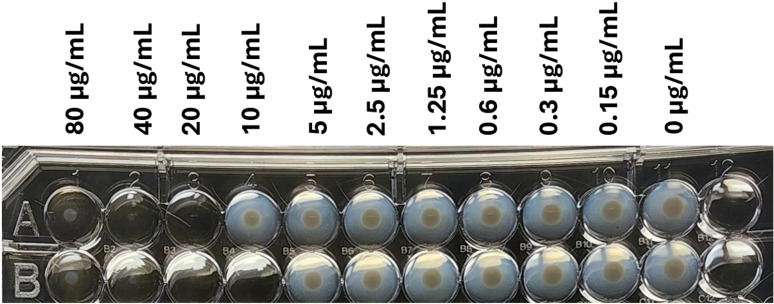
A photograph of the microwell plate used for microdilution test after overnight incubation.

We first determined the minimum inhibitory concentration (MIC) of the batch of AgNbO_3_ particles used throughout the present work, by assessing its effect on the growth of *Escherichia coli* cells in the presence of the particles suspended into the microwells at different concentrations as described in the method section. The result of one test replicate is presented in Fig 5 and as it can be seen, the MIC is somewhere in the 10–20 µg/mL range. Over three runs with two replicates each, the MIC fluctuated between 10 and 20 µg/mL.

Visual detection of growth and determination of MIC in the presence of interfering compounds, such as hemoglobin, can be difficult. So, subculture from wells onto liquid culture medium was used to confirm the result of MIC determination by visual reading. We observed a significant increase in the MIC, corresponding to loss of antimicrobial activity of the particles, with increasing concentration of both hemoglobin and hemin (Table 1). The inhibitory effect of hemoglobin was found to be higher than that of hemin. This difference is even more apparent when using the molar concentrations of the compounds: Each hemoglobin molecule reduces the antimicrobial activity ~500 times more than a molecule of hemin. As there are only four times more heme moiety on hemoglobin compared to hemin, we concluded that the heme may not be responsible for the inhibitory effect by hemoglobin. It should be stressed that the reduction in the antimicrobial activity by hemoglobin is not specific to AgNbO_3_ particles, rather it is generic to silver. Indeed, when carrying the test with the AgNO_3_ salt in the presence of hemoglobin, we similarly noted an increase in the AgNO_3_ MIC against *Escherichia coli*. These observations, combined, demonstrate that free hemoglobin antagonizes the antimicrobial activity of silver compounds with a mechanism where the heme moiety is not the determinant factor.

**Table 1:** The MIC values of AgNbO_3_ particles and AgNO_3_ against *Escherichia coli* for different concentrations of hemoglobin and hemin. To convert to molar concentrations, we have used the molar masses of 651.94 g/mol and 65000 g/mol, respectively, for hemin and hemoglobin.

Hemoglobin				Hemin			
Con. (mg/mL)	Con. (µM)	MIC AgNbO_3_ (µg/mL)	MIC AgNO_3_ (µg/mL)	Con. (mg/mL)	Con. (µM)	MIC AgNbO_3_ (µg/mL)	MIC AgNO_3_ (µg/mL)
0	0.00	16	8	0	0.00	16	8
1.25	1.92	32	256	1.25	0.02	32	32
2.50	3.83	64	256	2.50	0.04	32	32
5.00	7.67	128	256	5.0	0.08	32	32
10.0	15.34	128	128	10.0	0.15	64	32
20.0	30.68	256	256	20.0	0.31	64	32

The use of AgNbO_3_ particles in the presence of hemoglobin released from lysed red blood cells is likely to fail when treating bacterial infections. Therefore, mitigation strategies, ideally involving safe additives to the particles should be investigated. We began with iron due to the ease of inclusion of iron on the particles and on promising findings in the literature, reporting the enhancing effect of iron on conventional antimicrobials [36].

### 3.2 The impact of iron on the antimicrobial activity of AgNbO_3_ particles

The effect of iron ions on the viability of bacterial cells has been studied extensively. Iron, in particular the soluble ferrous form Fe^2+^, is a good electron donor and thus well suited and used as an essential element of the respiratory chain of bacteria cells [37]. Pathogenic bacteria in particular have developed sophisticated systems to acquire sufficient levels of iron as they need iron for the expression of virulence factors and for survival in the host system where ferrous iron (Fe^2+^) is seldom available. The host system, which is for the most part aerobic, allows the presence of the oxidized ferric form Fe^3+^. Even then, iron is not freely available, as, in humans for example, two thirds of iron is sequestered into erythrocytes as heme bound to the oxygen carrying protein hemoglobin [38].

Pathogenic bacteria acquire iron from the host using two mechanisms; The first involves direct contact between the bacterium and the source of iron and the subsequent removal of the iron by its reduction and uptake. The second and the more common, is the synthesis of high-affinity compounds that chelates iron from its source [39]. Such compounds are named siderophores, of which there are 500 types and without exception all of them have a higher affinity for Fe^3+^ [40]. Siderophore-iron complexes or other iron-binding compounds may then enter the bacterium through specialized high-affinity receptors [41]. Once inside the cell cytoplasm, the iron is reduced to Fe^2+^ and spontaneously released. Elevated levels of Fe^2+^, in combination with H_2_O_2_ (A byproduct of aerobic metabolism or of interaction with silver compounds) can contribute to oxidative stress via the production of reactive oxygen species (ROS) in the form of radical hydroxides through the Fenton reaction [42]:


$$\begin{array}{c} \text{F}{\text{e}}^{2+}+{\text{H}}_{2}{\text{O}}_{2}\to \text{F}{\text{e}}^{3+}+\text{O}{\text{H}}^{--}{+}^{•}OH \end{array}$$

To elucidate the potential mechanism responsible for combinatorial effect of AgNbO_3_ particles and iron ions, we studied the surface charge and elemental composition of the particle surface with more detail, as discussed in the supporting information (see Fig A and Table A of S5 Appendix). We have also demonstrated the generation of ROS in *Escherichia coli* as a result of its interaction with AgNbO_3_ particles using 2’, 7’-dichlorodihydrofluorescein diacetate (H_2_DCFDA) dye which has been detailed in our prior publication [8]. The measurement method is presented in the supporting information section (S6 Appendix). Briefly, upon entry of the dye to the live cells, acetate groups of the dye are cleaved by intracellular esterases. Once a nonfluorescent reduced form of dye is oxidized by reactive oxygen intermediates, it is converted to highly fluorescent DCF, whose fluorescence is proportional to the level of intracellular ROS. The test result has shown that the ROS concentrations (As measured by relative fluorescent units or RFU) are at background level for the tested AgNbO_3_ concentrations of 0, 4, and 8 µg/mL. However, at the AgNbO_3_ MIC concentration of 16 µg/mL, the amount of ROS increases from the background level by a statistically significant amount.

We next tested the MIC shifting tests using different concentrations of FeCl_3_ and FeSO_4_ in between 0.1 and 100 µM. FeSO_4_ was selected to test the possible effects of Fe^2+^ according to the Fenton reaction. On the other hand, FeCl_3_ dissociates to Fe^3+^, which isn’t expected to exert influence on the AgNbO_3_ antimicrobial activity as the Fenton reaction is irreversible [43]. The determined MIC in the presence of either FeCl_3_ or FeSO_4_ is presented in Table 2. As it is noted, despite expectations, the presence of Fe^2+^, a reactant for the Fenton reaction, worsened the antimicrobial activity of AgNbO_3_ by two-folds. In contrast, Fe^3+^ improved antimicrobial activity by two-folds. That said, the influence of either iron salts on the AgNbO_3_ MIC may not be as significant as the reported synergism of silver/transition metals of up to 8-fold against certain bacterial species [17].

**Table 2:** The MIC values of AgNbO_3_ particles against *Escherichia coli* at the presence of different concentrations of FeCl_3_ and FeSO_4_.

FeCl_3_		FeSO_4_	
Conc. (µM)	MIC (µg/mL)	Conc. (µM)	MIC (µg/mL)
0.1	16	0.1	16
1	8	1	32
10	8	10	32
100	8	100	32

Still, we thought that the presence of Fe^3+^ could play a part in the mechanism of antimicrobial action of AgNbO_3_ given that there was enhancement by factor of 2 in the AgNbO_3_ activity when supplying it with Fe^3+^ ions. Based on the empirical observation, we hypothesized that after inoculation, genes responsible for cellular iron recruitment and metabolism are induced for optimal exponential growth [44]. The presence of silver-containing AgNbO_3_ subsequently perturbs this process. We should note that iron in the form of Fe^3+^ is already present on the particles due to transfer from balls during the ball milling process as can be noticed from XPS spectra presented and discussed in detail in supplementary data (see Fig B of S5 Appendix). So, we decided to see that if further increasing the iron content of the AgNbO_3_ particles can improve the antimicrobial activity, thereby compensating for the activity loss in the presence of blood products encountered in physiological media. We synthesized AgFe_0.05_Nb_0.95_O_3_ particles and repeated experiments on the antimicrobial activity by the micro broth dilution assay and on the surface of different plates. Unfortunately, there was neither a noticeable change in the value of MIC, nor any observable compensation on the lost activity of the particles on the chocolate agar plate (CAP). Perhaps, the amount of iron on the original AgNbO_3_ particles, transferred via impact with steel balls during its preparation (see Table A of S5 Appendix) has already plateaued the likely enhancement in the antimicrobial activity and adding extra iron on the particles could no longer significantly improve the activity.

### 3.3 Testing the ability of K_2_EDTA to restore the antimicrobial activity of AgNbO_3_ particles

Inspired by the work of Gnanadhas et al [15], who demonstrated the use of capping agents to shield silver nanoparticles from the inhibitory effects of serum proteins, we looked for an alternative approach that could work using similar mechanisms to mitigate the antagonizing effect of Hemoglobin from lysed red blood cells on the antimicrobial activity of AgNbO_3_ particles. In this regard we took the long known EDTA stimulation seen in hemolysates due to the formation of an iron-EDTA complex [45] as a cue. Interestingly, the published results have illustrated no adverse effect of EDTA on the antimicrobial activity of silver. For instance, Freddi et al [46] have successfully prepared wool–EDTA–Ag complex displaying prominent antimicrobial activity against bacteria.

An important characteristic of EDTA in the context of our investigation is related to its interaction with the bacterial membrane structure. Our model organism *Escherichia coli*, as with all Gram-negative bacteria, possess an outer membrane enriched with lipopolysaccharides (LPS), which serve as a structural framework for the bacterial envelope. The stability of this outer layer is maintained by divalent cations, particularly Ca^2+^ and Mg^2+^, which form salt bridges between the negatively charged phosphate groups of LPS molecules [47]. The presence of these cations are also crucial in mitigating the electrostatic repulsion between the polyanionic LPS molecules. Exposure to EDTA, a potent chelator, removes these stabilizing cations, leading to outer membrane destabilization and the likelihood of increased susceptibility to the antimicrobial effects of AgNbO_3_. The extent of this perturbation was demonstrated by Marvin et al., who reported that EDTA treatment led to the release of up to 40% of LPS and outer membrane proteins in *Escherichia coli*, though the degree of this effect was strain dependent [48].

For Gram-positive bacteria which lack LPS and therefore any structural reliance on Ca^2+^ and Mg^2+^ for an individual cell, EDTA can still provide a synergistic role with antibacterial agents due to their anti-biofilm properties [49,50]. EDTA acts as a permeating and sensitizing agent for treating biofilm-associated conditions. The structural integrity and resilience of biofilms are attributed to extracellular polymeric substances (EPS), which are a complex mixture of polysaccharides, proteins, nucleic acids and lipids [51]. Cations such as Ca^2+^ and Mg^2+^ act as links between the polysaccharides providing the biofilm its structural rigidity. The chelating effects of EDTA reduces the concentration of these cations in the EPS thereby dissolving the biofilm and enabling direct interactions between the antimicrobial agent and bacteria cells. This mechanism was employed by Said et al. to explain the ability of a wound dressing consisting of silver bound to EDTA and benzethonium chloride capable of a three log reduction in viable cells of a biofilm [52]. The result was a great improvement over silver containing dressing without EDTA which caused at most one log reduction of viable bacteria in the model biofilm.

**Fig 6:**
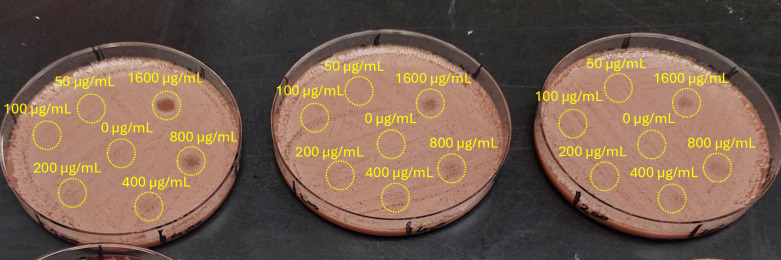
Effect on antimicrobial activity of K_2_EDTA on the antimicrobial activity of AgNbO_3_ particles on CAP plates. The concentrations of AgNbO_3_ particles on each spot are provided in the units of ng/mm^2^. K_2_EDTA amounts on the plates: from left to right: 1600, 800, and 400 µg on the whole surface area of the plate, respectively, corresponding to 0.25, 0.12 5, 0.0625 µg/mm^2^.

We therefore verified the ability of K_2_EDTA to mitigate the impact of lysed red blood cells on the antimicrobial activity of AgNbO_3_ particles. Results obtained with CAP plates are presented in Fig 6. Different amounts of K_2_EDTA were added prior to spreading bacterial cell suspension. The inclusion of K_2_EDTA at 0.25 µg/mm^2^ reduced the sMIC from >1600 µg/mL to 400 µg/mL, a 4-fold improvement.

The addition of K_2_EDTA also partially restored the inhibitory effect of lysed blood in the micro broth dilution susceptibility format. We utilized three types of broth media: MHB, MHB + %5 lysed blood, and MHB + 6 mg/mL hemoglobin, both in the absence and presence of 400 µg/mL of K_2_EDTA. The main observations are consistent with the results shown in Table 1. In this case, 6 mg/mL of hemoglobin increased the MIC of AgNbO_3_ particles by a factor of 8× while the shift by 5% lysed blood increased the MIC by 16-fold (See Table 3). Adding 400 µg/mL of K_2_EDTA to MHB had no noticeable effect on the MIC when lysed blood or hemoglobin is absent from the media, indicating that K_2_EDTA at this concentration neither inhibits nor augments the antimicrobial activity of the particles. However, the combination of presence of K_2_EDTA with AgNbO_3_ partly neutralizes the inhibitory action of lysed blood and hemoglobin on AgNbO_3_ activity with 4-fold reduction in MICs. The success of K_2_EDTA in improving the antimicrobial activity of nanostructured AgNbO_3_, despite the presence of lysed blood and isolated hemoglobin, suggest that the primary mechanism of inhibition caused by hemoglobin likely involves the direct sequestration of AgNbO_3_. Most likely, the main route of countering this inhibition by K_2_EDTA is to shield the AgNbO_3_ from hemoglobin exposure, because the role of EDTA in improving the antimicrobial activity of AgNbO_3_ was only seen in media containing either lysed blood or hemoglobin in isolation. Therefore, antimicrobial synergy between EDTA and AgNbO_3_ suggested by cell-wall breakdown is not a primary factor under the conditions we tested. The findings of this study regarding the use of K_2_EDTA can have significant implications for the application of AgNbO_3_ in wound dressings, where the presence of blood or its exudates can neutralize the antimicrobial activity of AgNbO_3_. In addition to the minimal risk of K_2_EDTA for human health [53], its independent ability to enhance antimicrobial activity, as demonstrated in this study, makes it a promising choice as an additive to silver-based medical products used in environments prone to blood contamination.

**Table 3:** The MIC values of AgNbO_3_ particles against *Escherichia coli* at the presence of lysed blood and hemoglobin, in the absence and presence of 400 µg/mL of K_2_EDTA.

	No EDTA			With 400 µg/mL of K_2_EDTA		
Media	MHB	MHB 5% Blood	MHB 6 mg/mL hemoglobin	MHB	MHB 5% Blood	MHB 6 mg/mL hemoglobin
**MIC (µg/mL)**	16	256	128	16	64	32

## 4. Conclusion

We demonstrated that while whole blood slightly inhibits the antimicrobial activity of AgNbO_3_ particles, the inhibition by lysed blood is stronger. Accordingly, we decided to test the antimicrobial activity of the particles in the presence of hemoglobin and hemin, which result from the lysis of blood cells. While both compounds exhibited strong inhibition, the inhibition per molecule of hemoglobin was over two orders of magnitude greater than that of a molecule of hemin. Therefore, we concluded that the heme moiety, common to the two compounds, was not the main determinant of the inhibition. Attempting to mitigate the inhibitory action of hemoglobin, we tested iron inclusion on the particles to no avail. By contrast, including K_2_EDTA proved successful in countering the inhibitory impact of hemoglobin.

## Supporting information

S1 Appendix Physical transformation of the AgNbO_3_ particles after subjecting them to low and high energy ball milling. (DOCX)

S2 Appendix Stability of AgNbO_3_ under harsh conditions. (DOCX)

S3 Appendix Measuring antimicrobial activity of AgNbO_3_ by broth microdilution method against 16 species of pathogenic bacterial and fungal species. (DOCX)

S4 Appendix Antimicrobial activity on different agar mediums against various bacterial species. (DOCX)

S5 Appendix The iron content of AgNbO_3_ particles transferred during ball milling. (DOCX)

S6 Appendix Methodology for illustrating the involvement of reactive oxygen species (ROS) in antimicrobial activity of AgNbO_3_ particles. (DOCX)
