# Supplementary material for: Identification and mitigation of blood’s interference with the antimicrobial activity of AgNbO3 particles
Source: PLoS One. 2025 Jun 24;20(6):e0313055. doi: 10.1371/journal.pone.0313055 (PMC12186951; doi:10.1371/journal.pone.0313055)
Supplement: S1 Appendix — (DOCX) [file pone.0313055.s001.docx]

# **S1 Appendix. Physical transformation of the AgNbO_3_ particles after subjecting them to low and high energy ball milling**

## **1. Methodology for transmission electron microscopy images**

The size and morphology of the particles were inspected using transmission electron microscopy (TEM). The microscope was a JEM-1230 electron microscope from JEOL, Ltd (Japan Electron Optics Laboratory) and the voltage used for the experiments was set at 80 KV. The copper grids, containing a carbon film, were from Electron microscopy Sciences (Pennsylvania, USA). 5 µL of the suspension were added onto the grid. The sample was given 1 min to settle. A paper filter was used to remove extra liquid from the sample. The sample was given a few days to dry as much as possible.

## **2. Results and discussion**

The synthesis route for AgNbO_3_ is initiated by the ceramic method, where the precursor metal oxides Ag_2_O and Nb_2_O_5_ are involved in a direct reaction to yield perovskite phase AgNbO_3_ crystals [1]. In the high energy ball milling (HEBM) step, these crystals were confined within an oscillating crucible and subjected to repeated collisions with metallic balls. The resulting impact forces fracture the crystals. Crystal fracture is believed to increase the density of edges (Interface between two different facets of the perovskite crystals) and corners that are found at the intergranular boundaries of the as-milled brittle perovskite crystals [2]. In contrast to the bulk crystal, atoms or ions located at edges and corners do not have their full coordination numbers and often give rise to the perovskite’s chemically reactive properties [3]. As seen in the Fig A presentation, one notable visual difference between AgNbO_3_ crystals at the end of the ceramic process (Untreated AgNbO_3_) and those subjected to HEBM is the color transformation from bright yellow to black. This indicates a strong light absorption over the visible spectrum, likely to be induced by a change in the perovskite’s band gap, which could suggest a strong influence on its chemical reactivity according to the literature [4]. That said, particles after HEBM usually exhibit lower than theoretical specific surface area, due to agglomeration, masking access to the chemically reactive grain boundaries. For this reason, the low energy ball milling (LEBM) process is used to apply a shear stress on the agglomerates, being sufficiently energetic to de-agglomerate the particles, but not energetic enough to cause cold welding and allow the re-agglomeration. All these processes combined make up the activation process and provide the treated AgNbO_3_ its antimicrobial characteristics.


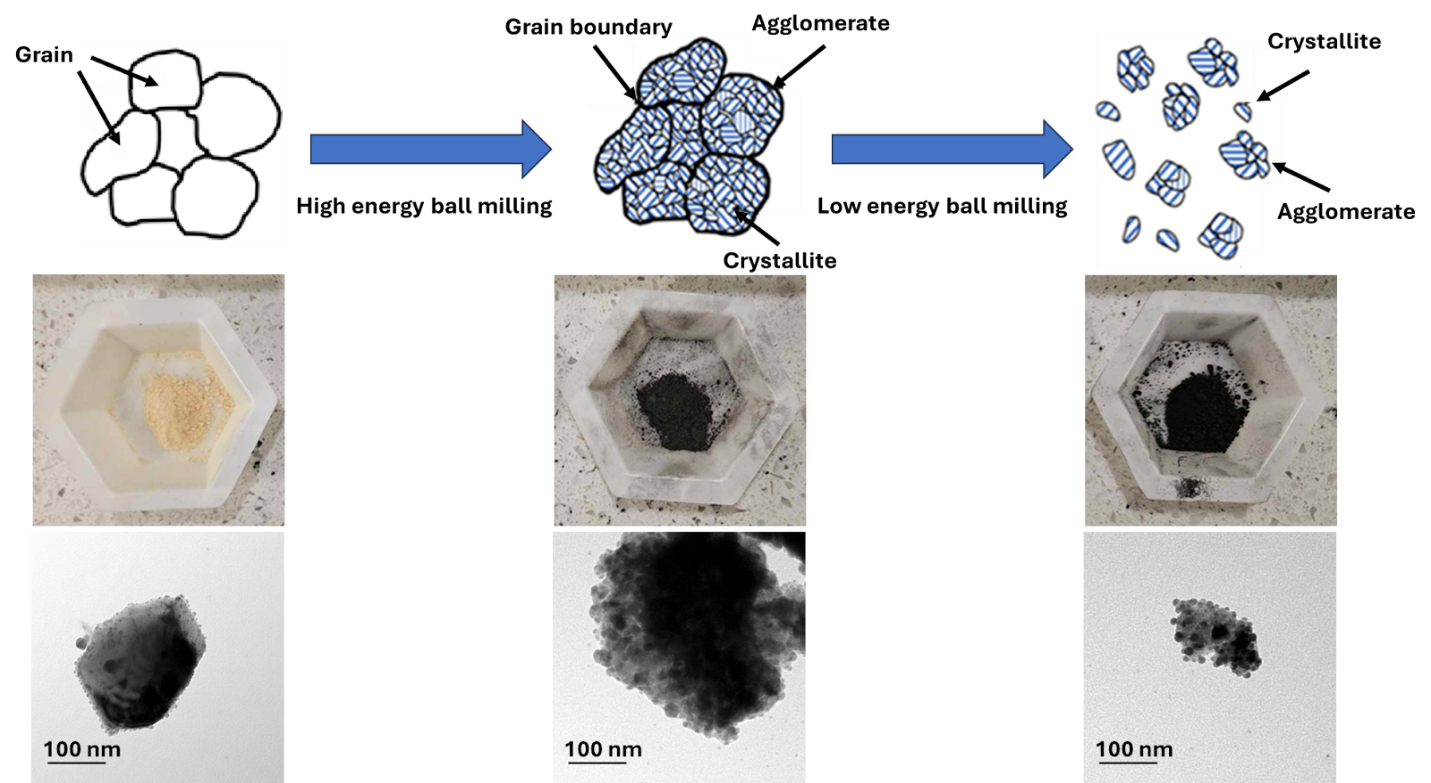


**Fig A. Physical transformation of the AgNbO_3_ particles after subjecting them to low and high energy ball milling.** The nanoscale changes in crystal structure are schematically presented in the first row. The second row presents the visual appearance of the powder, which is a qualitative indication of light absorption bandwidth. In the third row TEM image of a randomly selected particle is presented.

## **3. References**

(1) Valant M, Axelsson AK, Zou B, Alford N. Oxygen transport during formation and decomposition of AgNbO_3_ and AgTaO_3_. *Journal of materials research* 2007, 22 (6), 1650-1655. doi: 10.1557/JMR.2007.0196

(2) Alamdari H, Royer S. Mechanochemistry. In Perovskites and related mixed oxides: concepts and applications; *John Wiley & Sons* 2015, pp. 30-42. doi: 10.1002/9783527686605

(3) Klabunde KJ, Richards RM. Nanoscale materials in chemistry; *John Wiley & Sons* 2009, pp. 7-8.

(4) Fernández-García M, Martínez-Arias A, Hanson JC, Rodriguez JA. Nanostructured Oxides in Chemistry: Characterization and Properties. *Chemical reviews* 2004, 104 (9), 4063−4104. doi: 10.1021/cr030032f
