# Supplementary material for: Identification and mitigation of blood’s interference with the antimicrobial activity of AgNbO3 particles
Source: PLoS One. 2025 Jun 24;20(6):e0313055. doi: 10.1371/journal.pone.0313055 (PMC12186951; doi:10.1371/journal.pone.0313055)
Supplement: S2 Appendix — (DOCX) [file pone.0313055.s002.docx]

# **S2 Appendix. Stability of AgNbO_3_ under harsh conditions**

The stability and corrosion resistance of AgNbO_3_ nanoparticles were assessed by measuring long-term silver ion release in extremely basic, acidic, saline and bleach environments. Briefly, 1 L of each media was poured into 1 L glass beakers (Two beakers for each media). Three 10 mL replicate samples were taken from each of the 1 L beakers and marked as blanks for the respective media. Next, 500 mg of either AgNbO_3_ or Ag_2_O was added to one beaker of each media, which were covered to prevent evaporation or effects of light. At time points (2, 7, 14, 21, 28, 35) days, three 10 mL replicate samples were taken from each beaker and 30 mL of solution was added to the beakers to compensate for the removed volume. The collected samples were analyzed for silver ion concentration by Avio 200 ICP-OES (PerkinElmer). The obtained values of the ICP measurement were based on a calibration curve drawn using AgNO_3_ with Ag^+^ equivalent of 1-100 ppm and set to record the emission line for Ag^+^, at 328 nm. As already mentioned, each sample measurement was performed in three replicates, and the values were averaged. The results, summarized in Table A, showed minimal Ag⁺ release under these conditions, relative to the Ag_2_O nanoparticle, indicating that AgNbO₃ is chemically stable in harsh environments.

**Table A. Raw data for silver ion released from a 500 mg sample of either AgNbO_3_ or Ag_2_O within 1 L of any of the following media: Distilled water, basic NaOH solution (pH = 9), acidic HCl solution (pH = 4), salt solution with 3.5% NaCl and bleach solution with 0.5% NaClO.**

|  | Ag^+^ concentration (mg/L) | | | | | | | | | |  |
| --- | --- | --- | --- | --- | --- | --- | --- | --- | --- | --- | --- |
|  | Distilled water | NaOH solution (pH = 9) | HCl solution (pH = 4) | NaCl solution (3.5% NaCl) | NaClO solution (0.5% NaClO) | Distilled water | NaOH solution (pH = 9) | HCl solution (pH = 4) | NaCl solution (3.5% NaCl) | NaClO solution (0.5% NaClO) | |
| Background | 0.16 | 0.16 | 0.16 | 0.17 | 0.17 | 0.16 | 0.16 | 0.16 | 0.16 | 0.21 | |
| Storage time (d) | AgNbO_3_ (500 mg/L) | | | | | Ag_2_O (500 mg/L) | | | | | |
| 2 | 1.50 | 1.09 | 1.03 | 0.33 | 0.45 | 12.56 | 12.32 | 8.72 | 0.38 | 0.36 | |
| 7 | 1.89 | 1.48 | 1.11 | 0.32 | 0.45 | 20.67 | 22.56 | 19.57 | 0.31 | 0.38 | |
| 14 | 2.40 | 1.92 | 1.28 | 0.32 | 0.45 | 36.01 | 39.46 | 41.38 | 0.31 | 0.40 | |
| 21 | 1.90 | 1.63 | 1.02 | 0.11 | 0.22 | 43.80 | 48.92 | 57.60 | 0.23 | 0.27 | |
| 28 | 2.22 | 1.44 | 1.10 | 0.10 | 0.20 | 48.94 | 55.95 | 61.40 | 0.11 | 0.24 | |
| 35 | 2.38 | 1.82 | 1.13 | 0.10 | 0.19 | 51.64 | 57.55 | 64.93 | 0.11 | 0.23 | |
