# Supplementary material for: Identification and mitigation of blood’s interference with the antimicrobial activity of AgNbO3 particles
Source: PLoS One. 2025 Jun 24;20(6):e0313055. doi: 10.1371/journal.pone.0313055 (PMC12186951; doi:10.1371/journal.pone.0313055)
Supplement: S3 Appendix — (DOCX) [file pone.0313055.s003.docx]

# **S3 Appendix. Measuring antimicrobial activity of AgNbO_3_ by broth microdilution method against 16 species of pathogenic bacterial and fungal species**

The antimicrobial activity of AgNbO_3_ nanoparticles against common pathogens were assessed employing broth microdilution antimicrobial susceptibility test (AST), which involves growing bacterial cells inside a series of wells on a microwell plate containing growth media. Each well was supplied with different concentrations of the antimicrobial agent, differing by a factor of two from one well to the next. Known number of bacterial cells in the range of 10^5^ CFU (CFU = colony forming unit, meaning a cell that is viable and can divide) was dispensed into each well. After overnight incubation, the wells were inspected for signs of growth by visual inspection or turbidimetry. Thus, the minimum concentration required to inhibit growth was determined and reported as minimum inhibitory concentration (MIC) value. Table A below illustrates the MIC value of the typical nanostructured AgNbO_3_ powder against 18 pathogens encountered in arthroplasty infections.

**Table A. The MIC values of the typical nanostructured AgNbO_3_ powder against common pathogens.**

| **Pathogen** | **MIC (µg/mL)** |
| --- | --- |
| *Staphylococcus epidermidis* | 32 |
| *Staphylococcus aureus* | 4-8 |
| *Staphylococcus lugdunensis* | 16 |
| *Enterococcus faecalis* | 64 |
| *Pseudomonas aeruginosa* | 4 |
| *Escherichia coli* | 4-8 |
| *Streptococcus agalactiae* | 32 |
| *Streptococcus mitis/oralis* | 8-16 |
| *Corynebacterium striatum* | 8 |
| *Staphylococcus caprae* | 8 |
| *Staphylococcus capitis* | 4 |
| *Cutibacterium acnes* | 16 |
| *Candida parapsilosis* | 8 |
| *Candida albicans* | 4 |
| *Serratia marcescens* | 32 |
| *Enterobacter cloacae complex* | 32 |
