# Supplementary material for: Identification and mitigation of blood’s interference with the antimicrobial activity of AgNbO3 particles
Source: PLoS One. 2025 Jun 24;20(6):e0313055. doi: 10.1371/journal.pone.0313055 (PMC12186951; doi:10.1371/journal.pone.0313055)
Supplement: S4 Appendix — (DOCX) [file pone.0313055.s004.docx]

# **S4 Appendix. Antimicrobial activity on different agar mediums against various bacterial species**

## **1. Methodology**

Four types of agar plates obtained from Becton Dickinson, including Muller Hinton Agar (MHA), McConkey (MAC), Blood Agar Plate (BAP) and Chocolate Agar Plate (CAP), were used to assess the impact of their composition on the antimicrobial activity of microbial cells.

The surface of each plate was streaked using a swab soaked in *Escherichia Coli* (ATCC 25922) cell suspension (1/100 of 0.5 McFarland). Then 5 µL of AgNbO_3_ with different concentrations (50, 100, 200, 400, 800, 1600 µg/mL) was added onto the surface of each plate at locations corresponding to 2, 4, 6, 8, 10 and 12 O’clock respectively). Plates were incubated at 35 ˚C overnight. Plates were analyzed for bacterial growth.


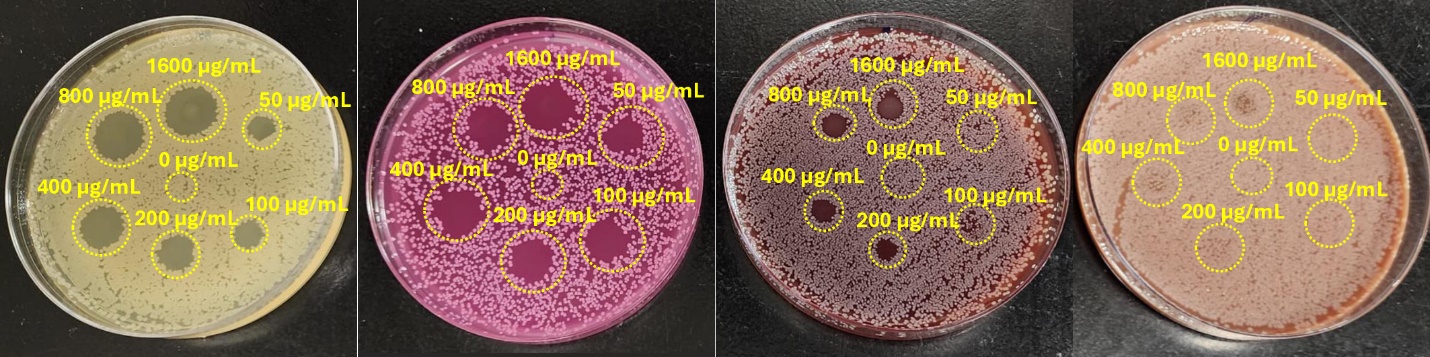


**Fig A. From left to right, respectively, MHA, MAC, BAP, and CAP.** Full antimicrobial activity and inhibition of bacterial growth is seen on MHA and MAC plates for all AgNbO_3_ concentrations. Partial inhibition of AgNBO_3_ antimicrobial activity is seen on BAP plate, and complete inhibition of activity on CAP plate.

We used a different approach to show the antimicrobial activity of AgNbO_3_ on MHA, MAC, BAP and CAP. Each plate was divided in half (top and bottom). First, 150 µL of AgNbO_3_ (500 µg/mL) was added to the top part and allowed to dry. The test strains included *Klebsiella pneumoniae* (ATCC 700603), *Pseudomonas aeruginosa* (ATCC 27853) and *Escherichia Coli* (ATCC 25922). For each tested strain, a cell suspension (50 µL from a 1/100 dilution of 0.5 McFarland) was added to the top and bottom section on each plate and spread using a sterile loop. Plates were incubated overnight at 35 ˚C and subsequently photographed.

## **2. Results and discussion**

As seen in Figs A-C, a full inhibition of bacterial growth, for all tested strains, is seen in MHA and MAC plates (Media without blood). The antibacterial activity of AgNbO_3_ was partially inhibited on blood containing BAP and completely inhibited on lysed blood containing CAP. The observed inhibition of AgNbO_3_ activity by hemoglobin is equally seen against *Klebsiella pneumoniae* (ATCC 700603) and *Pseudomonas aeruginosa* (ATCC 27853) as it is with our model strain *Escherichia Coli* (ATCC 25922). Given that both *Klebsiella pneumoniae* (ATCC 700603) and *Pseudomonas aeruginosa* (ATCC 27853) are well known for their significant role in hospital acquired infections [1, 2], these findings highlight a potential limitation of AgNbO_3_ in clinical applications where blood exposure is unavoidable.


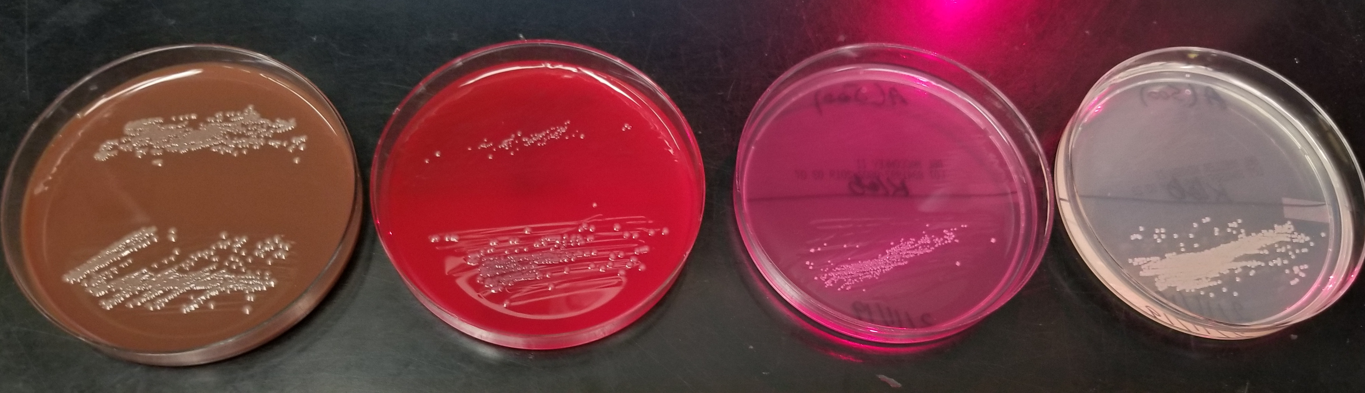


**Fig B. Antimicrobial activity of AgNbO_3_ particles against *Klebsiella pneumoniae* on different plates. From left to right, respectively, MHA, MAC, BAP, and CAP.** Full inhibition of bacterial growth is seen on MHA and MAC plates, partial inhibition on BAP plate, and complete inhibition on CAP plate.


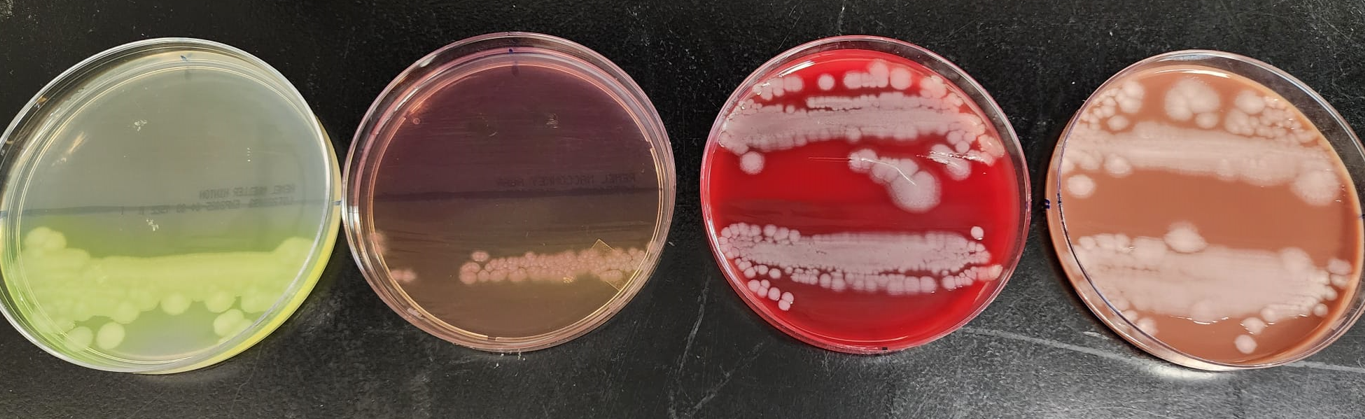


**Fig C. Antimicrobial activity of AgNbO_3_ particles against *Pseudomonas aeruginosa* on different plates. From left to right, respectively, MHA, MAC, BAP, and CAP.** Full inhibition of bacterial growth is seen on MHA and MAC plates, partial inhibition on BAP plate, and complete inhibition on CAP plate.


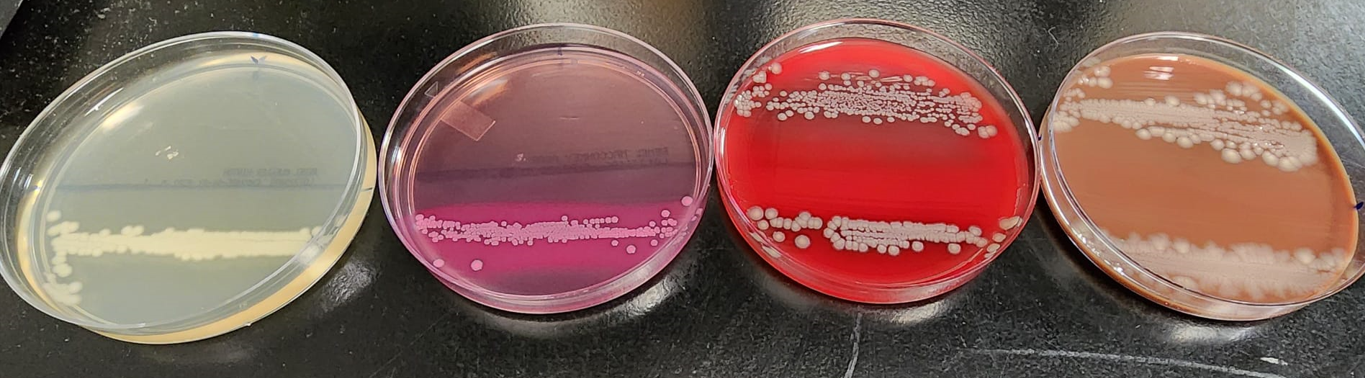


**Fig D. Antimicrobial activity of AgNbO_3_ particles against *Escherichia Coli* on different plates. From left to right, respectively, MHA, MAC, BAP, and CAP.** Full inhibition of bacterial growth is seen on MHA and MAC plates, partial inhibition on BAP plate, and complete inhibition on CAP plate.

## **3. References**

(1) Phan S, Feng H, Huang R, Lee ZX, Moua Y, Phung OJ, Lenhard JR. Relative Abundance and Detection of *Pseudomonas aeruginosa* from Chronic Wound Infections Globally. *Microorganisms* 2023, 11 (5), 1-14. doi: 10.3390/microorganisms11051210

(2) Lin X, Li C, Zhang S, Yang, X, Jiang M. The Global and Regional Prevalence of Hospital-Acquired Carbapenem-Resistant *Klebsiella pneumoniae* Infection: A Systematic Review and Meta-analysis. *Open forum infectious diseases* 2024, 11 (2), 1-15. doi: 10.1093/ofid/ofad649
