# Supplementary material for: Identification and mitigation of blood’s interference with the antimicrobial activity of AgNbO3 particles
Source: PLoS One. 2025 Jun 24;20(6):e0313055. doi: 10.1371/journal.pone.0313055 (PMC12186951; doi:10.1371/journal.pone.0313055)
Supplement: S5 Appendix — (DOCX) [file pone.0313055.s005.docx]

# **S5 Appendix. The iron content of AgNbO_3_ particles transferred during ball milling**

The XPS spectrum of AgNbO_3_ particles is presented in Fig A. The spectra present are associated with different orbitals of elements Ag, Nb, O, Fe, and C. The most resolved peaks from each of these elements, that is Ag 3d, Nb 3d, O 1s, Fe 2p and C 1s, were integrated and corrected through their respective elemental sensitivities (Provided by the elemental library tied to the XPS machine) to give the atomic percentages. These are presented in Table A along with their corresponding values for the same powder, which was obtained before performing ball milling for increasing antimicrobial activity.


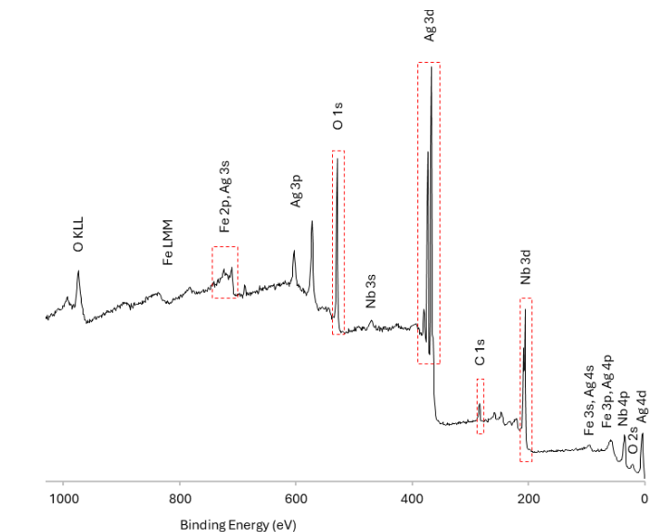


**Fig A. The XPS spectrum of nanostructured AgNbO_3_ particles.** The most well-resolved peaks from each of the element present on the sample surface (Ag, Nb, O, Fe and C) are indicated in red dashed-square regions.

**Table A. The percentage of atomic constituents of AgNbO_3_ particles before and after performing ball milling, as inferred from the XPS.**

| **Element** | **% Before ball milling** | **% After ball milling** |
| --- | --- | --- |
| Ag | 19.46 | 19.28 |
| Nb | 20.64 | 14.89 |
| O | 41.66 | 45.29 |
| Fe | Not detected | 3.30 |
| C | 18.53 | 17.23 |

A particular remark in relation to the data is the higher proportion of Ag compared to Nb (19.48% vs 14.89%) despite their presumed stoichiometric 1:1 ratio. This relates to the phenomena that between both cations (Ag, Nb) of the AgNbO_3_ perovskite structure, the larger one has the lowest surface energy and is therefore more likely to segregate itself towards the exterior in order to minimize the overall surface energy of the crystal [1, 2]. Two other elements were determined, the first being typically occurring adventitious C, the other being Fe. The transfer of Fe contaminants towards the AgNbO_3_ surface occurs during the ball milling process of the Activated Reactive Synthesis (ARS) method, due to the erosion of the steel balls when shearing against fine AgNbO_3_ powder inside aqueous media. 1-4 wt % Fe have been reported to be normally present in most powders milled with steel grinding medium, although the magnitude of contamination may rise with time and intensity of milling [3].

Fig B presents the deconvoluted high-resolution spectra of Ag 3d, Nb 3d, O 1s and Fe 2p. The corresponding origin of the deconvolutions are assigned on the basis of the information provided from the reference guidebook of standard spectra for intensification and interpretation of XPS data [4]. In the case of Ag 3d, the spectrum presents two peaks related to the Ag 3d_5/2_ and Ag 3d_3/2_ core energy levels, at 368.48 and 374.53 eV respectively. These values align closely to the line positions of 368.3 and 374.3 eV. A corresponding set of Ag 3d_5/2_ shoulders at lower binding energies of 366.28 and 365.03 eV is also observed, this relates to the Ag^+^ oxidic state, where Ag is bonded to O at the AgNbO_3_ surface level in the form of Ag_2_O [5]. Similarly to Ag 3d, the spectrum of Nb 3d may be split into Nb 3d_5/2_ and Nb 3d_3/2_ core energy levels, due to spin coupling. The respective binding energies are 207.3 and 210.0 eV, much higher than the 202.4 and 205.1 eV suggested by the guidebook. This shift of approximately 5 eV for both core energy levels of Nb 3d arises from the fact that Nb is in an Nb^5+^ oxidation state, coordinated with oxygen in an octahedral geometry [6].


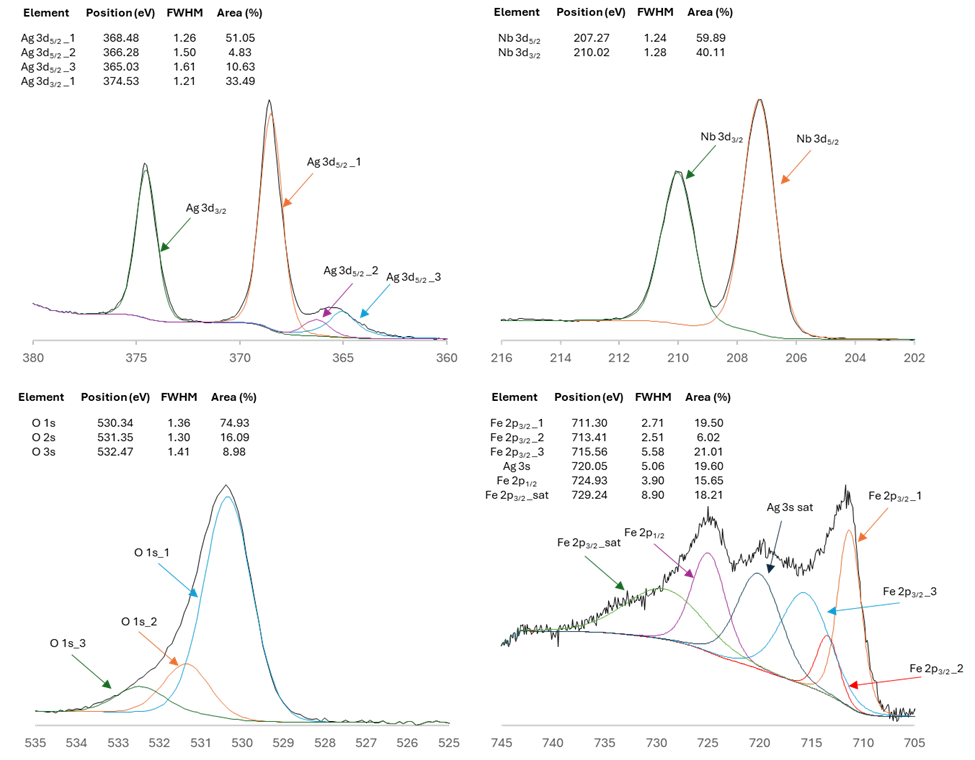


**Fig B Deconvoluted peaks of Ag 3d, Nb 3d, O 1s and Fe 2p.**

In regards to the O 1s spectrum, it can be deconvoluted into three peaks at binding energies of 530.3, 531.3 and 532.5 eV. The principal O 1s_1 peak at 530.3 eV can arise from structural oxygen bound to Ag and Nb while the accompanying O 1s_2 and O 1s_3 peaks are produced by adsorbed oxygen [7]. Another likely explanation is that O 1s_2 and O 1s_3 peaks at 531.3 and 532.5 eV can be sourced from contributions of metal oxide reduction with the atomic ratio of O/Ag or O/Nb at less than one, alluding to the possibility of oxygen vacancy formations [8].

As it is seen from Table A, all Fe atoms have been incorporated into the particles during the ball milling procedure. This aligns with the hypothesis that milling involves the use of steel balls and crucibles which break down and transfer Fe onto the particles. These transferred atoms can be split into Fe 2p_3/2_ and Fe 2p_1/2_ core energy levels, at respectively 711.3 and 724.9 eV. This is a likely outcome of Fe being in a Fe^3+^ oxidation state [9]. The Fe 2p_3/2_ peak has been further split to several components only to make a best fit in the deconvolution. According to the literature, Fe^3+^ will additionally manifest Fe 2p_3/2_ satellite peaks [10], one of which is covered by the Ag 3s peak and the other appearing at 729.2 eV according to Fig B. Referring to Table A, although a decrease in the atomic percentages of both Ag and Nb are seen after milling, the biggest decrease is seen in Nb. So, it could be concluded that, at least at the surface level, iron substitution occurs at the more catalytically active B-sites of the AgNbO_3_ perovskite.

## **References**

(1) Liu J, Kim JK, Wang Y, Kim H, Belotti A, Koo B.; et al. Understanding and mitigating A-site surface enrichment in Ba-containing perovskites: a combined computational and experimental study of BaFeO_3_. *Energy & Environmental Science* 2022, 15(10), 2069-2081. doi: 10.1039/d2ee01813f

(2) Higgins D, Wette M, Gibbons BM, Siahrostami S, Hahn C, Escudero-Escribano M, et al. Copper Silver Thin Films with Metastable Miscibility for Oxygen Reduction Electrocatalysis in Alkaline Electrolytes. *ACS Applied Energy Materials* 2018, 1 (5), 1995. doi: 10.1021/acsaem.8b00090

(3) Suryanarayana C. Mechanical alloying and milling. *Progress in Materials Science* 2001; 46, 136-143.

(4) Moulder JF, Stickle WF, Sobol PE, Bomben KD. Handbook of X-ray photoelectron spectroscopy; Perkin-Elmer Corporation, 1992.

(5) Parashar PK, Komarala VK. Engineered optical properties of silver-aluminum alloy nanoparticles embedded in SiON matrix for maximizing light confinement in plasmonic silicon solar cells. *Scientific reports* 2017, 7(1), 2-3. doi: 10.1038/s41598-017-12826-1

(6) Ofoegbuna T, Darapaneni P, Sahu S, Plaisance C, Dorman JA. Stabilizing the B-site Oxidation State in ABO_3_ Perovskite Nanoparticles. *Nanoscale* 2019, 11(30), 7. doi: 10.1039/x0xx00000x

(7) Skryleva EA, Kubasov IV, Kiryukhantsev-Korneev PV, Senatulin BR, Zhukov RN, Zakutailov KV, et al. XPS study of Li/Nb ratio in LiNbO_3_ crystals. Effect of polarity and mechanical processing on LiNbO_3_ surface chemical composition. *Applied Surface Science* 2016, 389, 387-394. doi: 10.1016/j.apsusc.2016.07.108

(8) Gao R, Li Z, Zhang X, Zhang J, Hu Z, Liu X. Carbon-dotted defective CoO with Oxygen Vacancies: A synergetic design of bifunctional cathode catalyst for Li-O_2_ batteries. *ACS Catalysts* 2016, 6, 402-403. doi:10.1021/acscatal.5b01903

(9) He G, Liu X, Li R, Zhai D, Liu Y, Xie C, et al. Silver modified Ba_1-x_Co_0.7_Fe_0.2_Nb_0.1_O_3-δ_ perovskite performing as a cathodic catalyst of intermediate-temperature solid oxide fuel cells. *ACS Applied Materials & Interfaces* 2020, 12, 9425-9426. doi: 10.1021/acsami.9b19634

(10) Pereira A, Cros A, Delaporte PH, Marine W, Sentis M. XeCl laser treatment of steel surface. *Applied Surface Science* 2002, 845-850.
