# Supplementary material for: Identification and mitigation of blood’s interference with the antimicrobial activity of AgNbO3 particles
Source: PLoS One. 2025 Jun 24;20(6):e0313055. doi: 10.1371/journal.pone.0313055 (PMC12186951; doi:10.1371/journal.pone.0313055)
Supplement: S6 Appendix — (DOCX) [file pone.0313055.s006.docx]

# **S6 Appendix. Methodology for illustrating the involvement of reactive oxygen species (ROS) in antimicrobial activity of AgNbO3 particles**

The intracellular ROS accumulation in *Escherichia coli* cells exposed to AgNbO_3_ particles was measured using cell permeant 2’, 7’-dichlorodihydrofluorescein diacetate (H_2_DCFDA) dye (Invitrogen by Thermo Fisher Scientific, Canada). Upon entry into the live cells, acetate groups are cleaved by intracellular esterases. The nonfluorescent reduced form of dye is oxidized by ROS and, converted to highly fluorescent DCF, whose fluorescence is proportional to the level of intracellular ROS. Briefly, the *Escherichia coli* bacteria cells were adjusted to a McFarland 0.5 standard and then diluted 1:100 in 150 mL Lb in a 500 mL flask. The cultures were grown to the onset of exponential phase with an optical density (OD_600_) of approximately 0.12, then nanostructured AgNbO_3_ particles were added at a final concentration of 4, 8 and 16 µg/mL. Absence of nanostructured AgNbO_3_ particles and H_2_O_2_ (1 mM concentration) were used as negative and positive controls respectively. 1 mL aliquots were collected at times 30, 60 and 90 min following the additions of nanostructured AgNbO_3_ particles and H_2_O_2_. The aliquots were then centrifuged and washed once with phosphate buffered saline (PBS, pH = 7.2). The H_2_DCFDA dye was added to a final concentration of 10 μM and incubated for 1 h at 37°C in the dark. The labeled cells were washed once and resuspended in 500 μL of PBS. The fluorescence signal of a 200 μL aliquot was analyzed using a Victor fluorometer (Perkin-Elmer, Waltham, MA, USA) at 485 nm excitation and 535 nm emission wavelengths. Results were expressed as relative fluorescence units (RFU) and were normalized according to the number of live cells present. Three independent experiments were performed to enhance the validity of the results.
